# Dual mini-fragment plate fixation for Neer type-II and -V distal clavicle fractures

**DOI:** 10.1097/OI9.0000000000000078

**Published:** 2020-08-14

**Authors:** Michael J. Chen, Brett P. Salazar, Julius A. Bishop, Michael J. Gardner

**Affiliations:** Department of Orthopaedic Surgery, Stanford University Medical Center, Redwood City, CA

**Keywords:** distal clavicle, dual mini-fragment plating, implant prominence

## Abstract

Contemporary methods for open reduction and internal fixation of displaced distal clavicle fractures have excellent rates of union and high rates of reoperation for symptomatic implant removal. The authors describe their preferred surgical technique and case series of patients with Neer Type-II and -V distal clavicle fractures treated with lower profile dual mini-fragment plates using interdigitating screws placed into the distal segment to enhance fixation.

## 1 Introduction

Nonoperative management of displaced distal clavicle fractures has a 37% risk of nonunion and a 40% risk of malunion.^[1]^ Similar to the deforming forces affecting midshaft clavicle fractures, the weight of the arm pulls the shoulder girdle inferiorly in combination with the pull of surrounding musculature to generate 3-dimensional deformity.^[2]^ The resulting deformity likely adversely affects spontaneous healing and eventual shoulder girdle kinematics.

Open reduction and internal fixation of displaced distal clavicle fractures has a high success rate of achieving union and restores normal scapulothoracic mechanics.[3][4][5][6] Hook plates are useful for maintaining reduction, but limit postoperative rehabilitation and often require removal to reduce implant prominence and rotator cuff irritation.[7][8] Distal clavicle-specific precontoured plates allow for locking screw fixation into the distal fragment without adverse effects of spanning the acromioclavicular (AC) joint.[3][5][9] However, these plates may not fit every patient's anatomy and implant prominence still carries a reoperation risk as high as 52% for removal.^[9]^

As previously described for fixation of midshaft clavicle fractures, dual mini-fragment plating of distal clavicle fractures allows for better customized fitting of implants to bone, and creates a lower profile construct when compared with precontoured plates.[10][11] When placed in orthogonal planes, mini-fragment plates provide adequate stability to maintain fracture reduction to healing. In this article, we present our surgical technique for fixation of Neer type-II and -V distal clavicle fractures using orthogonally placed dual mini-fragment plates with interdigitating locking screws placed into the distal segment.

## 2 Authors’ preferred surgical technique

### 2.1 Preoperative examination

A physical exam is initially performed to exclude limb-threatening injuries. Scapula and chest wall injuries frequently occur with clavicle fractures and should be identified. Standard radiographic evaluation includes an upright anterior-posterior (AP) cephalic tilt view of the clavicle and an axillary view of the shoulder to identify any horizontal displacement, which may be difficult to appreciate on the AP view. Computed tomography scans have little influence in guiding management and are not required.

### 2.2 Operating room setup

The patient is positioned supine on a reversed radiolucent cantilever table with a Plexiglas arm board supporting the injured extremity. Fluoroscopy is brought in from the opposite side of the bed and tilted cranially and caudally to obtain “inlet” and “outlet” views of the shoulder girdle, respectively.

### 2.3 Surgical technique

A longitudinal incision in the line of the clavicle is made anterosuperiorly over the distal clavicle and AC joint through skin and subcutaneous tissues. Traversing supraclavicular nerves are identified and protected if able. The fascia of the anterior deltoid is incised and the deltoid carefully elevated off the anterior distal clavicle in an extraperiosteal fashion, thus completing exposure of the fracture. The AC joint is identified by palpation and careful dissection of the distal clavicle fragment. The AC capsule is left undisturbed.

For fracture patterns involving a single distal fragment (Neer type-II), the distal end can be reduced to the proximal shaft with strategic pointed reduction clamp placement on either side of the fracture. Provisional fixation is maintained with either the clamp or Kirscher (K) wires. If the distal fragment is large enough and the fracture line is oblique, interfragmentary compression between the medial and lateral segments is obtained with a 2.0 or 2.4 mm lag screw. A 2.4 mm T- or Y-shaped plate (Smith & Nephew, Memphis, TN) is then applied to the superior surface of the distal clavicle and provisionally fixed. A fluoroscopic view is checked to confirm the precise position of the plate in relation to the AC joint. Multiple locking screws are placed into the distal fragment and cortical screws into the clavicle diaphysis. Provisional fixation can then be removed if the reduction is stable. Next, a supplemental 2.0, 2.4, or 2.7 mm straight plate (Smith & Nephew, Memphis, TN) is selected, cut to appropriate length, and contoured to the anterior surface. The plate is typically secured to the proximal segment first with nonlocking screws. Next, as many anterior to posterior screws as possible are placed into the distal fragment, “interdigitating” in an orthogonal fashion with the locking screws of the superior plate to increase construct stability. An implant that allows for variable angle locking can be particularly useful in this situation.

**Figure 1:**
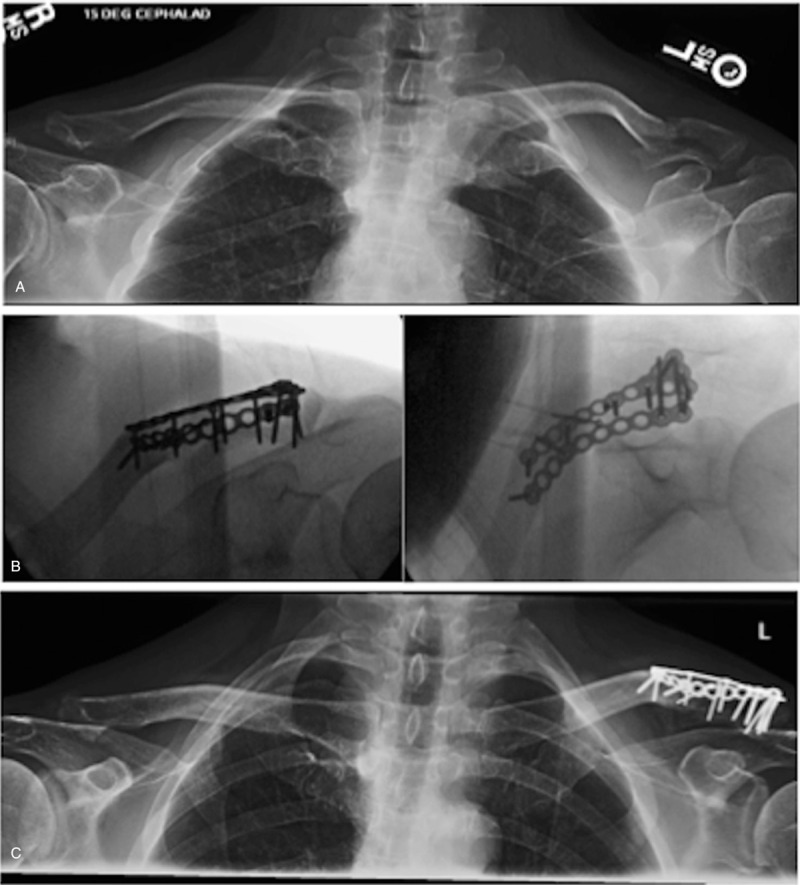
(A) Injury radiographs, (B) intraoperative fluoroscopy, and (C) final postoperative radiographs demonstrating healing of a Neer type-V distal clavicle fracture fixed with dual mini-fragment plates. Multiple lag screws were placed into the inferior fragment attached to the coracoclavicular ligaments. Note the interdigitating screws placed through orthogonal plates to enhance fixation in the distal segment.

**Figure 2:**
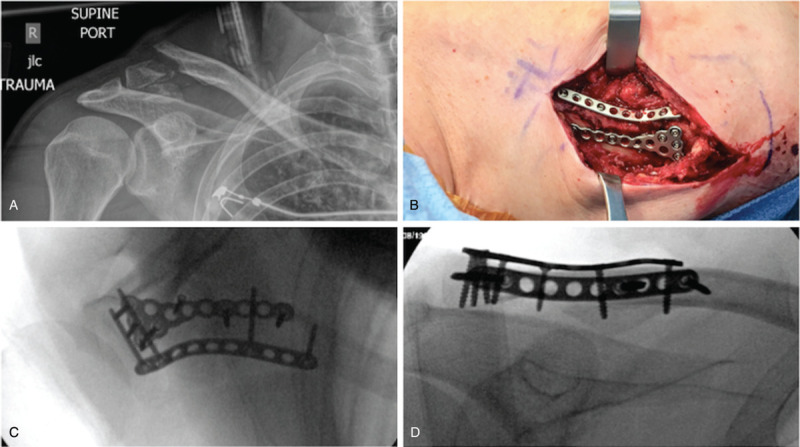
(A) Injury radiographs of another Neer type-V distal clavicle fracture. (B) An intraoperative photograph showing definitive fixation with a Y-shaped mini-fragment plate on the superior distal clavicle surface, and a straight mini-fragment plate contoured to the anterior distal clavicle surface. (C, D) Final fluoroscopic images demonstrating anatomic reduction and the dual mini-fragment plated construct with interdigitating locking screws in the distal fragment. A single lag screw prior to plate application was placed into the inferior fragment attached to the coracoclavicular ligaments.

For comminuted fracture patterns with a large inferior fragment attached to the coracoclavicular ligaments (Neer type-V), the inferior fragment is reduced and clamped to either the proximal or distal segment, followed by interfragmentary compression with a small diameter lag screw when possible. The reduction of medial and lateral segments and plate fixation then proceeds as detailed above. In comminuted patterns not amenable to clamp reduction and lag screw fixation, pointed reduction clamps are used to control the entire medial segment of the clavicle. If the lateral segment is large and robust enough, it can also be controlled with a clamp; otherwise, the clamp can be applied to the acromion to allow for manipulation. Length, alignment, and rotation can then be restored. Provisional fixation can then be achieved with K-wires inserted through the acromion and across the fracture if needed. The fracture is then bridged with “interdigitating” fixation into the distal fragment as mentioned before (Figs. 1 and 2).

Suspensory augmentation can be considered when stable interlocking fixation into the distal segment cannot be achieved secondary to comminution or an extremely small distal segment. After reduction and plating of the fracture, the base of the coracoid process is exposed by blunt dissection inferior to the distal clavicle. Retractors are placed on either side of the coracoid protecting the neurovascular structures traversing from medial to lateral beneath. A suture anchor is placed into the base of the coracoid in a cranial-caudal direction. One suture limb is brought around the posterior aspect of the clavicle and tied to the other limb over the plates once reduction of the fracture and CC interspace has been obtained. This completes fixation.

Postoperatively, patients are allowed to use the injured extremity for activities of daily living with a 2-pound weight lifting restriction. Restrictions are removed at 6 weeks and return to sport typically occurs around 3 months postsurgery.

## 3 Results

After institutional review board approval, a retrospective review was performed of all patients over the last 3 years with distal clavicle fractures treated surgically at our institution by the orthopaedic trauma service. Of the initial 19 patients identified, 8 were treated with dual mini-fragment plates. Two patients were excluded due to lack of follow-up leaving 6 patients available for final review.

See Table 1 for patient characteristics and outcomes. All patients had achieved union, as defined by radiographic bridging bone, without loss of reduction or fixation. Maintenance of the reduction was defined as a lack of interval displacement of the distal fragment relative to the proximal clavicle. Average age at the time of surgery was 46 years (range 24–63 years). One patient (17%) was female. Average body mass index (BMI) was 24.1 (range 17.4–30.5). Four patients had Neer type-II fracture patterns and 2 had Neer type-V patterns. Two patients (33%) had associated injuries to the chest wall and/or shoulder girdle. Average QuickDASH score at final follow-up was 5 (range 0–11.4). One patient (17%) underwent elective implant removal after healing due to symptomatic implants. This patient in particular had a low BMI of 17.4 and slender profile. There were no other complications, including infection or wound problems. Average follow-up was 38 weeks (range 16–60 weeks).

**Table 1:**
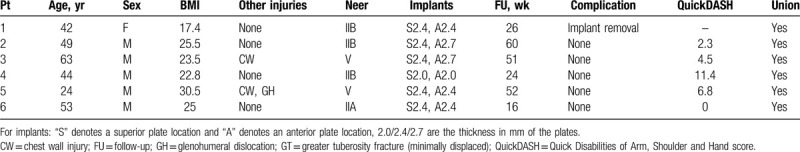
Demographic data, injury characteristics, and postoperative outcomes of patients with distal clavicle fractures treated with dual mini-fragment plates.

## 4 Discussion

While precontoured distal clavicle-specific and hook plates can reliably maintain reduction of these difficult-to-treat fractures, both implants have limitations and often require reoperation for implant removal.[7][9] Mini-fragment plates offer the advantage of being lower profile, and can be sized and contoured to match anatomy on an individual basis. Compared with contemporary techniques, dual mini-fragment plating appears to provide enough mechanical stability to achieve healing without vascular compromise. This is evident as all of the patients in this series proceeded to uneventful union. Paying careful attention to placing orthogonal interlocking screws into the small distal segment is a critical aspect of this technique to gain stable fixation.

An alternative technique to be considered that utilizes lower profile fixation is suspensory fixation. Yagnik et al^[12]^ recently reported on 21 patients with unstable distal clavicle fractures who underwent stabilization with cortical button fixation and CC ligament reconstruction with a 100% union rate and minimal complications. More studies are needed before this technique can be recommended over plate fixation.

One patient in our limited series developed implant irritation and underwent subsequent removal. This patient had a slender physical profile and low BMI, which likely factored into implant prominence despite the relative low profile of the construct. This illustrates that any metal implants used for fixation in this location have the potential to cause prominence and soft-tissue irritation. A larger study is needed to determine the actual rate of removal, success of healing, and patient-reported outcomes when using dual mini-fragment plates for open reduction and internal fixation of distal clavicle fractures, and how it compares to that of larger precontoured plates.

Further limitations to our study include that we did not have documented shoulder range of motion to report. However, because these injuries do not involve the glenohumeral joint, shoulder stiffness is uncommon and has rarely been encountered in the senior author's experience. One of our patients had less-than-ideal follow-up of 8 weeks. However, this patient had demonstrated healing of the fracture without loss of reduction or fixation, and was included in this series to help illustrate the effectiveness of this technique for achieving stable and durable fixation.

## 5 Conclusion

Distal clavicle fractures present unique challenges to the orthopaedic traumatologist when treated surgically. Dual mini-fragment plates contoured specifically to the anatomy of individual patients create a sound construct and offer the advantage of being lower profile with more customization compared with contemporary techniques. Interlocking screws in the distal fragment enhances stability to maintain reduction to healing.
